# The Impact of Metabolic Syndrome Risk Factors on Lung Function Impairment: Cross-Sectional Study

**DOI:** 10.2196/43737

**Published:** 2023-09-05

**Authors:** Rafael Molina-Luque, Guillermo Molina-Recio, Domingo de-Pedro–Jiménez, Carlos Álvarez Fernández, María García–Rodríguez, Manuel Romero-Saldaña

**Affiliations:** 1 Estilos de Vida, Innovación y Salud Instituto Maimónides de Investigación Biomédica de Córdoba (IMIBIC) Córdoba Spain; 2 Departamento de Enfermería, Famarcología y Fisioterapia Universidad de Córdoba Córdoba Spain; 3 Indorama Ventures Química Sociedad Limitado Unipersonal Polígono Industrial Guadarranque San Roque, Cádiz Spain; 4 Departamento de Salud y Seguridad del Trabajo Ayuntamiento de Córdoba Córdoba Spain; 5 Departamento de Enfermería y Nutrición Facultad de Ciencias Biomédicas y de la Salud Villaviciosa de Odón Madrid Spain

**Keywords:** cardiometabolic risk factor, lung function, metabolic syndrome, restrictive lung disease, spirometry

## Abstract

**Background:**

Metabolic syndrome (MetS) is a constellation of risk factors increasingly present in the world’s population. People with this syndrome are at an increased risk of cardiovascular disease and type 2 diabetes mellitus. Moreover, evidence has shown that it affects different organs. MetS and its risk factors are independently associated with impaired lung function, which can be quantified through spirometric variables.

**Objective:**

This study aims to determine whether a high number of MetS criteria is associated with increased lung function decline.

**Methods:**

We conducted a descriptive cross-sectional study with a random sample of 1980 workers. Workers with acute respiratory pathology (eg, influenza), chronic respiratory pathology (eg, chronic bronchitis), or exposure to substances harmful to the lungs (eg, organic and inorganic dust) were not included. MetS was established based on harmonized criteria, and lung function was assessed according to spirometric variables. On the basis of these, classification into restrictive lung disease (RLD), obstructive lung disease, and mixed lung disease (MLD) was performed. In addition, the association between MetS and lung function was established based on analysis of covariance, linear trend analysis, and multiple linear regression.

**Results:**

MetS was associated with worse lung function according to all the spirometric parameters analyzed (percentage of predicted forced expiratory volume in 1 second: mean 83, SD 13.8 vs mean 89.2, SD 12.8; *P*<.001 and percentage of predicted forced vital capacity: mean 85.9, SD 11.6 vs mean 92, SD 11.3; *P*<.001). Moreover, those diagnosed with MetS had a higher prevalence of lung dysfunction (41% vs 21.9%; *P*<.001), RLD (23.4% vs 11.2%; *P*<.001), and MLD (7.3% vs 2.2%; *P*<.001). Furthermore, an increasing number of MetS criteria was associated with a greater impairment of pulmonary mechanics (*P*<.001). Similarly, with an increasing number of MetS criteria, there was a significant linear trend (*P*<.001) in the growth of the prevalence ratio of RLD (0 criteria: 1, 1: 1.46, 2: 1.52, 3: 2.53, 4: 2.97, and 5: 5.34) and MLD (0 criteria: 1, 1: 2.68, 2: 6.18, 3: 9.69, and 4: 11.37). Regression analysis showed that the alteration of all MetS risk factors, adjusted for various explanatory variables, was significantly associated with a worsening of spirometric parameters, except for forced expiratory volume in 1 second/forced vital capacity.

**Conclusions:**

The findings have shown that an increase in cardiometabolic risk factors is associated with a more significant worsening of spirometric variables and a higher prevalence of RLD and MLD. As spirometry could be a crucial tool for monitoring patients at risk of developing chronic pathologies, we conclude that this inexpensive and easily accessible test could help detect changes in lung function in patients with cardiometabolic disorders. This highlights the need to consider the importance of cardiometabolic health in lung function when formulating public health policies.

## Introduction

### Background

The impact of noncommunicable chronic diseases worldwide is increasingly important. Their prevalence and social and economic impact have increased in the recent years, placing them at the center of public health interest [1-4].

Among the most worrying pathological conditions, metabolic syndrome (MetS) stands out because it is a risk factor for cardiovascular disease and type 2 diabetes mellitus (DM), which are, in turn, the noncommunicable chronic diseases with the most significant impact worldwide [5,6]. MetS, according to the harmonized definition, is characterized by the comorbid presence of ≥3 of the following risk factors: dysglycemia, increased blood pressure, hypertriglyceridemia, abdominal obesity, and altered high-density lipoprotein (HDL) cholesterol levels [6]. The prevalence of MetS exceeds 30% in several countries and is expected to increase in the coming years [7,8]. All these make MetS one of the public health challenges of the 21st century; therefore, early detection is essential to avoid associated complications, even at an early age [9,10].

### Lung Dysfunction and MetS

In addition to cardiovascular pathologies and type 2 DM, MetS has been associated with alterations in other systemic organs, most notably alterations in lung function [11,12]. For example, researchers have observed that MetS is associated with the worsening of parameters measuring pulmonary mechanics, leading to respiratory diseases (asthma, chronic obstructive pulmonary disease, pulmonary hypertension, etc) [12-14]. Several MetS-related lung pathologies are currently the most prevalent, have significant morbidity and mortality, and are a major public health concern [15,16]. This link could be explained by insulin resistance, one of the main pathophysiological mechanisms of MetS, although its involvement in the modification of pneumocyte function is not yet clear [17-19].

Parameters for pulmonary function status are obtained through spirometry, which is the test of choice to assess lung function [20]. It is simple to execute for those who are well instructed and is routinely performed by health care professionals, for example, in primary care or during health examinations of workers [21,22]. A comparison of the values obtained in the test with the theoretical values, estimated from validated formulas (according to age, sex, height, etc), provides information on the lung condition [23,24]. It is, therefore, an essential public health tool for primary, secondary, and tertiary prevention of pathologies with a high impact on the population [25].

In this context, health care professionals, who play an essential role in disease prevention and health promotion, have a tool that, when used efficiently, would help to detect problems beyond pulmonary deterioration [26]. Regarding the latter, given that the onsets of MetS is reflected in spirometric variables, and its risk factors also do so independently, it is relevant to know whether a progressive deterioration of lung function is caused by the individual presence of the risk factors characterizing MetS. Thus, through public health programs (hospitals, communities, workplaces, etc) that include the study of these parameters from the perspective of cardiometabolic alterations, it would be possible to detect the risk factors and treat them early to prevent the development of MetS.

Therefore, the researchers hypothesized that the increase in the number of MetS components is associated with a greater decline in spirometric parameters and, therefore, an alteration in lung function. On this basis, the objective of this study was to determine how spirometric variables are affected because of the increase in the number of the MetS risk factors present.

## Methods

### Design, Population, and Sample

A descriptive cross-sectional study was conducted in the working population of the Cordoba City Council (Spain). The minimum sample size estimated was 1685 workers based on an expected prevalence of MetS of 14.9%, a power of 80%, a precision of 1.5%, and a confidence of 95%. The sample was randomly selected from workers who underwent a health examination between 2015 and 2019 at the occupational health unit of the City Council. All employees of the Cordoba City Council (blue-collar workers and white-collar workers) undergo an annual occupational health test. Each of the measurements carried out is stored in a computer program of the occupational health unit, which allows for the subsequent extraction of anonymized data. For this study, the number of workers selected was increased by 20% over the minimum sample size calculated to compensate for losses owing to noncompliance with the eligibility criteria.

Workers who were diagnosed with any acute respiratory pathology (influenza, common cold, etc) or chronic pulmonary disease (chronic bronchitis, emphysema, pneumonia, chronic obstructive pulmonary disease, etc) and those who could not perform the spirometry test according to the protocol were excluded from the study. In addition, the occupational risk of the positions held by the workers was assessed, and it was confirmed that there was no exposure to organic and inorganic dust or substances of high molecular weight that could lead to occupational lung pathologies or affect, transiently, the spirometry test results.

### Variables and Measures

Lung function was studied through spirometric parameters: forced expiratory volume in 1 second (FEV1; in L), forced vital capacity (FVC; in L), and the FEV1/FVC ratio. To determine the status of pulmonary function, the expected values of FEV1 and FVC were estimated and related to those obtained in spirometry by calculating the percentage of predicted FEV1 (FEV1%) and percentage of predicted FVC (FVC%) [24].

In addition, the percentage reductions of FEV1 and FVC with respect to the theoretical or expected values of FEV1 and FVC were calculated:

FEV1 reduction (%) = ([FEV1 – expected FEV1]/FEVI) × 100 **(1)**

FVC reduction (%) = ([FVC – expected FVC]/FVC) × 100 **(2)**

Workers were also categorized according to their dysfunctional pattern [27]: normal: FEV1/FVC>0.7 and FVC%>0.8; restrictive lung disease (RLD): FEV1/FVC>0.7 and FVC%<0.8; obstructive lung disease (OLD): FEV1/FVC<0.7 and FVC%>0.8; and mixed lung disease (MLD): FEV1/FVC<0.7 and FVC%<0.8. In addition, the aforementioned categories were grouped into a new dichotomous variable with the following values: normal and lung dysfunction (encompassing RLD, OLD, and MLD).

The spirometry test was performed using the DATOSPIR 120 C spirometer (Silbemed) and following the recommendations of the Spanish Society of Pneumology and Thoracic Surgery [27]. Workers were instructed not to use bronchodilators during the previous 6 to 48 hours (depending on the drug), to avoid caffeine during the previous 8 hours, not to smoke during the previous 24 hours, avoid alcohol and sedatives during the last 4 hours, and not to exercise during the previous 30 minutes. During the measurement, the worker wore a nose clip and was seated upright with his back against the backrest. Once the posture was correct, the worker was asked to inhale as much air as possible and then exhale as fast and hard as possible until they were told to stop. Workers had to perform at least 3 valid tests, with the highest spirometric values being chosen. The researchers considered a test valid when the test had a rapid start (back-extrapolated volume <5% of the FVC) and was performed without hesitation, involved continuous expiration for a duration not <6 seconds, had no abrupt end (last changes in volume lower than 0.025 L for ≥1 s), and had no anomalies in the technique (cough, new inhalation, among others). Overall, 2 or 3 acceptable maneuvers were necessary for spirometry test’s interpretation in which the difference between the 2 best FVC and FEV1 measures was not >0.2 L.

Moreover, risk factors included in the harmonized criteria for the diagnosis of MetS were considered: waist circumference (WC) ≥102 cm in men and ≥88 cm in women, triglycerides ≥150 mg/dL, HDL cholesterol <50 mg/dL in women and <40 mg/dL in men, blood pressure ≥130/85 mm Hg or being on treatment for high blood pressure and fasting glucose ≥100 mg/dL, being on antidiabetic treatment, or diagnosed with DM. Thus, the presence of ≥3 of these risk factors implies a diagnosis of MetS [6].

The independent variables collected were age (years), sex (female and male), smoking status (nonsmoker, ex-smoker, and smoker), physical activity level (light, moderate, and heavy), weight (kg), height (cm), BMI (kg/m^2^), WC (cm), hip circumference (cm), body fat percentage (Equation Córdoba for Estimation of Body Fat [28]), waist to hip ratio, waist to height ratio, systolic blood pressure (mm Hg), diastolic blood pressure (mm Hg), fasting plasma glucose (mg/dL), HDL cholesterol (mg/dL), and triglycerides (mg/dL). In addition, the diagnoses of DM (yes or no) and hypertension (yes or no) were collected.

Anthropometric variables were measured according to international recommendations [29]. Weight and height were collected using an Atlántida S11 stadiometer and scale (Básculas y Balanzas Añó-Sayol), with an accuracy of 0.1 kg and 0.1 cm, respectively. BMI was calculated based on these variables, and workers were categorized into normal weight, overweight, and obese groups, as proposed by the World Health Organization. WC was measured at end-expiration at the midpoint between the last rib and the iliac crest. Hip circumference was assessed at the most prominent point of the buttocks. Both variables were measured using a flexible tape, with the worker standing and their feet placed together. Blood pressure was measured according to the recommendations of the manual of arterial hypertension in primary care clinical practice [30], with the patients seated, using a calibrated digital sphygmomanometer (Omron M3, Omron Healthcare). All measurements were performed by specialized personnel to minimize the coefficient of variation. Each measurement was repeated 3 times, and the mean was calculated. Finally, physical activity was estimated in metabolic equivalents using the International Physical Activity Questionnaire.

### Ethics Approval

The study protocol complied with the Declaration of Helsinki for medical research involving human participants and was approved by the Andalusian Biomedical Research Ethics Committee (4427/Acta number 295).

### Informed Consent

All participants were informed, verbally and in writing, about the objectives of the health study. The researchers obtained informed consent following the current regulations.

### Statistical Analysis

Quantitative variables were presented as mean and SD, and qualitative variables were presented as absolute frequencies, percentages, and prevalence ratios. The goodness of fit of the quantitative variables to a normal distribution was studied using a Kolmogorov-Smirnov test with Lilliefors correction.

Hypothesis testing was performed with the 2-tailed Student *t* test for 2 means; *z* tests for independent proportions; and chi-square and Fisher exact tests, when necessary, for qualitative variables. In addition, linear trend tests, analysis of covariance, and multiple linear regression were used to determine the effect of other factors and covariates on the variation in spirometric parameters.

For all statistical analyses, an α error probability of <5% was accepted, and CIs were calculated at 95%. SPSS Statistics (version 22.0; IBM Corp) and EPIDAT 4.2 (Department of Sanidade, Xunta de Galicia) were used for statistical analysis.

## Results

### Description of the Sample

Of the 2069 randomly selected workers, 168 (8.12%) were excluded because they were diagnosed with an acute respiratory pathology or a chronic pulmonary pathology or were exposed to compounds that could alter their lung function, and in 41 (1.98%) other workers, it was impossible to assess MetS. Finally, 44 (2.13%) other workers were excluded because their clinical records did not include some spirometric variables. Thus, the final sample size was 1816.

The age of the sample ranged from 18.8 to 67 years. The prevalence of MetS was 18.3% (95% CI 16.5%-20.1%), being significantly higher in men (20.6%, 95% CI 18.5%-23.4%) than in women (16%, 95% CI 13.8%-18.5%; *P*<.001). BMI was higher among those with MetS (mean difference [MD] 7.1 kg/m^2^; *P*<.001). This group showed a higher prevalence of overweight and obesity (95.9% vs 49.3%; *P*<.001). In addition, in the MetS group, higher abdominal adiposity was observed according to the following parameters: (1) WC (MD 19.5 cm; *P*<.001), (2) waist to hip ratio (MD 0.1; *P*<.001), and (3) waist to height ratio (MD 0.12; *P*<.001).

All the other variables analyzed showed differences between those with and those without MetS, except for height (MD 0.2 cm; *P*=.73). Table 1 summarizes the main variables according to the presence or absence of MetS.

**Table 1:** Characteristics of the sample according to the presence of metabolic syndrome (MetS).

Variables			Total (n=1816)		With MetS (n=329, 18.1%)		Without MetS (n=1487, 81.9%)		*P* value^a^	
Age (years), mean (SD)			43.8 (10.7)		48.6 (9.5)		42.8 (10.7)		<.001	
**Sex, n (%)**										.01
	Female	919 (50.6)		146 (44.4)		773 (52)				
	Male	897 (49.4)		183 (55.6)		714 (48)				
Height (cm), mean (SD)			167.9 (9.3)		167.9 (9.3)		167.9 (9.4)		.98	
Weight (kg), mean (SD)			75.6 (17.3)		91.7 (16.7)		72.1 (15.3)		<.001	
**BMI (kg/m^2^), mean (SD)**			26.7 (5.4)		32.5 (5.3)		25.5 (4.5)		<.001	
	Underweight, n (%)	36 (2)		0 (0)		36 (2.4)				
	Normal weight, n (%)	731 (40.3)		13 (4)		718 (48.3)				
	Overweight, n (%)	635 (35)		95 (28.9)		540 (36.3)				
	Obesity, n (%)	414 (22.8)		221 (67.2)		193 (13)				
Waist (cm), mean (SD)			89.6 (14.1)		105.5 (11.2)		86 (12.1)		<.001	
Hip (cm), mean (SD)			102.7 (10.1)		111 (10.9)		100.9 (9)		<.001	
WHR^b^, mean (SD)			0.87 (0.097)		0.95 (0.09)		0.85 (0.09)		<.001	
WHtR^c^, mean (SD)			0.53 (0.081)		0.63 (0.07)		0.51 (0.07)		<.001	
Body fat (%), mean (SD)			31.4 (8.4)		38.9 (8.2)		29.8 (7.6)		<.001	
**Physical activity, n (%)**										.02
	Light	121 (6.7)		31 (9.4)		90 (6.1)				
	Moderate	457 (25.2)		93 (28.3)		364 (24.5)				
	Heavy	1238 (68.2)		205 (62.3)		1033 (69.5)				
**Smoking habit, n (%)**										<.001
	Nonsmoker	882 (48.6)		130 (39.5)		752 (50.6)				
	Ex-smoker	312 (17.2)		74 (22.5)		238 (16)				
	Smoker	620 (34.1)		124 (37.7)		496 (33.4)				
Glucose (mg/dL), mean (SD)			99.5 (28.1)		119.8 (46.8)		95.1 (19.3)		<.001	
HDL^d^ cholesterol (mg/dL), mean (SD)			63.2 (14.5)		54.8 (13.2)		65.1 (14.1)		<.001	
SBP^e^ (mm Hg), mean (SD)			123.7 (16.9)		137.4 (16.7)		120.6 (15.4)		<.001	
DBP^f^ (mm Hg), mean (SD)			76.9 (10.5)		86 (9)		74.9 (9.7)		<.001	
Triglycerides (mg/dL), mean (SD)			108.3 (68.8)		174.2 (101.3)		93.7 (48.4)		<.001	
HBP^g^, n (%)			704 (38.8)		277 (84.2)		427 (28.7)		<.001	
Type 2 DM^h^, n (%)			110 (6.1)		73 (22.2)		37 (2.5)		<.001	
FEV1^i^ (L), mean (SD)			3.1 (0.8)		2.8 (0.7)		3.1 (0.8)		<.001	
FEV1%^j^, mean (SD)			88.2 (13.2)		83 (13.8)		89.2 (12.8)		<.001	
FEV1 reduction^k^, mean (SD)			−16.3 (20.7)		−24.4 (24.4)		−14.5 (19.4)		<.001	
FVC^l^ (L), mean (SD)			4 (0.9)		3.7 (0.8)		4 (0.9)		<.001	
FVC%^m^, mean (SD)			90.9 (11.6)		85.9 (11.6)		92 (11.3)		<.001	
FVC reduction^n^, mean (SD)			−11.9 (15.2)		−18.7 (17.5)		−10.4 (14.3)		<.001	
FEV1/FVC, mean (SD)			0.77 (0.07)		0.76 (0.07)		0.78 (0.07)		<.001	
FEV1/FVC<0.7, n (%)			218 (12)		58 (17.6)		160 (10.8)		<.001	
FVC%<0.8, n (%)			300 (16.5)		101 (30.7)		199 (13.4)		<.001	
**Lung dysfunction, n (%)**			461 (25.4)		135 (41)		326 (21.9)		<.001	
	Restrictive lung disease	243 (13.4)		77 (23.4)		166 (11.2)		<.001		
	Obstructive lung disease	161 (8.9)		34 (10.3)		127 (8.5)		.30		
	Mixed lung disease	57 (3.1)		24 (7.3)		33 (2.2)		<.001		

^a^With MetS versus without MetS.

^b^WHR: waist to hip ratio.

^c^WHtR: waist to height ratio.

^d^HDL: high-density lipoprotein.

^e^SBP: systolic blood pressure.

^f^DBP: diastolic blood pressure.

^g^HBP: high blood pressure.

^h^DM: diabetes mellitus.

^i^FEV1: forced expiratory volume in 1 second.

^j^FEV1%: percentage of predicted forced expiratory volume in 1 second.

^k^FEV1 reduction (%) = ([FEV1 – expected FEV1]/FEV1) × 100.

^l^FVC: forced vital capacity.

^m^FVC%: percentage of predicted forced vital capacity.

^n^FVC reduction (%) = ([FVC – expected FVC]/FVC) × 100.

### MetS and Lung Function (Spirometric Parameters)

In relation to lung function, workers with MetS showed lower values of FEV1 (MD 0.3 L; *P*<.001), FVC (MD 0.32 L; *P*<.001), and FEV1/FVC ratio (MD 0.015; *P*<.001). Similarly, higher proportions of FEV1/FVC<0.7 (17.6% vs 10.8%; *P*<.001) and FVC%<0.8 (30.7% vs 13.4%; *P*<.001) were found among participants with MetS. This translated into the presence of MetS being associated with a higher prevalence of lung dysfunction (41% vs 21.9%; *P*<.001). Table 1 provides more detailed results.

### Individual MetS Criteria and Lung Function (Spirometric Parameters)

Several adjusted multiple linear regression models are presented in Table 2, where the dependent variables, spirometric variables, are represented in the rows, and the independent variables are represented in the columns. The adjusted multiple linear regression study confirms that a quantitative modification of the variables related to MetS criteria (increased WC, elevated plasma triglycerides, raised blood pressure, increased blood glucose, and decreased HDL) causes an alteration of the spirometric variables (Table 2). Specifically, it is observed that an increase in abdominal adiposity and insulin resistance–related variables lead to a significant decrease in FVC and FEV1. Finally, MetS was associated with reductions of 0.220 L and 0.277 L in FEV1 and FVC, respectively.

**Table 2:** Multiple linear regression for spirometric parameters (n=1816)^a^.

Variable			Waist		Glucose		HDL^b^		Triglycerides		High blood pressure		Metabolic syndrome
**FEV1^c^**													
	β	−.004		−.003		.003		−.001		−.078		−.220	
	Standardized β	−.065		−.116		.066		−.130		−.050		−.110	
	*t* test (*df*)	−3.728 (1809)		−7.410 (1809)		4.026 (1809)		−8.090 (1809)		−2.858 (1809)		−6.929 (1809)	
	*R*^2^ adjusted	0.554		0.564		0.554		0.566		0.552		0.562	
	*P* value	<.001		<.001		<.001		<.001		<.001		<.001	
**FEV1%^d^**													
	β	−.146		−.071		.096		−.034		−1.607		−6.046	
	Standardized β	−.156		−.152		.106		−.180		−.059		−.177	
	*t* test (*df*)	−6.186 (1809)		−6.597 (1809)		4.390 (1809)		−7.656 (1809)		−2.345 (1809)		−7.596 (1809)	
	*R*^2^ adjusted	0.059		0.061		0.049		0.069		0.042		0.069	
	*P* value	<.001		<.001		<.001		<.001		.02		<.001	
**FEV1 reduction^e^**													
	β	−.237		−.105		.127		−.051		−3.029		−9.147	
	Standardized β	−.162		−.142		.089		−.171		−.071		−.170	
	*t* test (*df*)	−6.397 (1809)		−6.201 (1809)		3.678 (1809)		−7.239 (1809)		−2.803 (1809)		−7.278 (1809)	
	*R*^2^ adjusted	0.055		0.054		0.041		0.061		0.038		0.061	
	*P* value	<.001		<.001		<.001		<.001		.005		<.001	
**FVC^f^**													
	β	−.004		−.004		.004		−.002		−.096		−.277	
	Standardized β	−.064		−.115		.067		−.121		−.050		−.115	
	*t* test (*df*)	−3.790 (1809)		−7.532 (1809)		4.184 (1809)		−7.714 (1809)		−2.977 (1809)		−7.390 (1809)	
	*R*^2^ adjusted	0.576		0.586		0.577		0.586		0.575		0.585	
	*P* value	<.001		<.001		<.001		<.001		<.001		<.001	
**FVC%^g^**													
	β	−.147		−.064		.096		−.029		−1.531		−5.941	
	Standardized β	−.178		−.154		.121		−.172		−.064		−.197	
	*t* test (*df*)	−7.016 (1809)		−6.640 (1809)		4.969 (1809)		−7.256 (1809)		−2.511 (1809)		−8.422 (1809)	
	*R*^2^ adjusted	0.045		0.042		0.032		0.047		0.022		0.056	
	*P* value	<.001		<.001		<.001		<.001		.01		<.001	
**FVC reduction^h^**													
	β	−.202		−.088		.124		−.038		−2.126		−7.966	
	Standardized β	−.187		−.163		.118		−.173		−.068		−.202	
	*t* test (*df*)	−7.365 (1809)		−7.041 (1809)		4.870 (1809)		−7.270 (1809)		−2.655 (1809)		−8.638 (1809)	
	*R*^2^ adjusted	0.047		0.045		0.031		0.046		0.022		0.057	
	*P* value	<.001		<.001		<.001		<.001		.008		<.001	
**FEV1/FVC**													
	β	−.004		−.006		−.003		−.005		−.055		−.099	
	Standardized β	−.009		−.023		−.007		−.052		−.004		−.006	
	*t* test (*df*)	−0.376 (1809)		−1.043 (1809)		−0.316 (1809)		−2.284 (1809)		−0.161 (1809)		−0.247 (1809)	
	*R*^2^ adjusted	0.126		0.127		0.126		0.129		0.126		0.126	
	*P* value	.71		.30		.75		.02		.87		.81	

^a^The variables in the rows are the dependent variables, and the variables in the columns are the independent variables. Models were adjusted by age, height, smoking habit, sex, and physical activity (dichotomized: light and active [moderate and heavy]).

^b^HDL: high-density lipoprotein.

^c^FEV1: forced expiratory volume in 1 second (L).

^d^FEV1%: percentage of predicted forced expiratory volume in 1 second.

^e^FEV1 reduction (%) = ([FEV1 − expected FEV1]/FEV1) × 100.

^f^FVC: forced vital capacity (L).

^g^FVC%: percentage of predicted forced vital capacity.

^h^FVC reduction (%) = ([FVC – expected FVC]/FVC) × 100.

### Number of MetS Criteria and Lung Function (Spirometric Parameters)

Table 3 shows the effect of the spirometric variables as a function of the number of MetS components.

**Figure 1:**
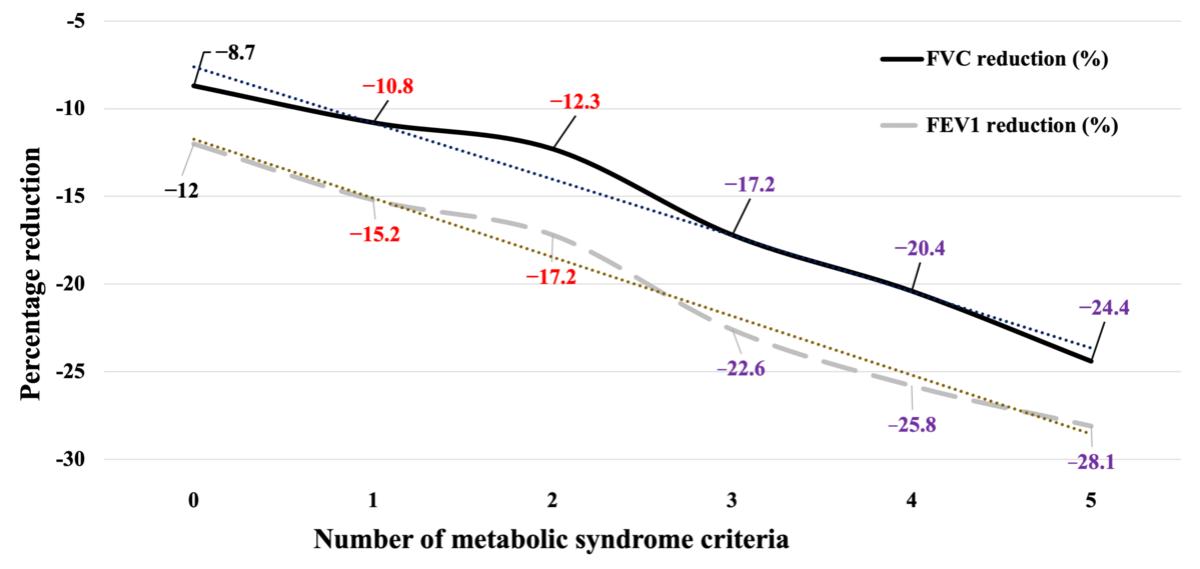
Percentage reductions of forced expiratory volume in 1 second (FEV1) and forced vital capacity (FVC) according to number of metabolic syndrome criteria. Same color means that there were no significant differences between values within a variable.

FVC and FVC% showed a decrease in values with an increasing number of MetS criteria. However, the differences found between having 1 or 2 criteria (FVC: MD 0.046 L, *P*=.16; FVC%: MD 1.330, *P*=.08; FVC reduction: MD 1.481%, *P*=.13), 3 or 4 criteria (FVC: MD 0.107 L, *P*=.06; FVC%: MD 2.066, *P*=.13; FVC reduction: MD 3.257%, *P*=.07), 3 or 5 criteria (FVC: MD 0.165 L, *P*=.15; FVC%: MD 4.322, *P*=.21; FVC reduction: MD 7.228%, *P*=.11), and 4 or 5 criteria (FVC: MD 0.058 L, *P*=.16; FVC%: MD 2.256, *P*=.53; FVC reduction: MD 3.971%, *P*=.40) were not significant in any of the variables studied. Nevertheless, it is worth noting that the percentage reduction of FVC from what was expected showed a clear linear downward trend, with an MD of 16 (SD 20) percentage points between those with 0 criteria and those with 5 criteria (*P*<.001; Figure 1). These results were also observed for FEV1, FEV1%, and FEV1 reduction. These findings indicate that an increase in the number of MetS criteria is associated with a significant reduction in lung function.

**Table 3:** Lung function according to the number of metabolic syndrome criteria.

Variables	Number of components, mean (SD)							*P* value^a^
	0 (n=540)	1 (n=554)	2 (n=393)	3 (n=223)	4 (n=95)	5 (n=11)		
FEV1^b^	3.16 (0.44)	3.09 (0.42)^c^	3.04 (0.44)^c^	2.93 (0.43)^d^	2.85 (0.44)^d^	2.77 (0.45)^d^	<.001	
FEV1%^e^	91 (13)	89.1 (12.7)^c^	87.5 (12.7)^c^	84 (12.8)^d^	81.6 (12.8)^d^	80.8 (12.7)^d^	<.001	
FEV1 reduction^f^	−12 (20)	−15.2 (20)^c^	−17.2 (20)^c^	−22.6 (20.1)^d^	−25.8 (20.1)^d^	−28.1 (20.1)^d^	<.001	
FVC^g^	4.07 (0.51)	3.99 (0.49)^c^	3.95 (0.5)^c^	3.78 (0.49)^d^	3.68 (0.5)^d^	3.62 (0.49)^d^	<.001	
FVC%^h^	93.5 (11.5)	91.8 (11.3)^c^	90.4 (11.3)^c^	86.7 (11.4)^d^	84.6 (11.4)^d^	82.3 (11.3)^d^	<.001	
FVC reduction^i^	−8.7 (14.8)	−10.8 (14.8)^c^	−12.3 (14.8)^c^	−17.2 (14.8)^d^	−20.4 (14.8)^d^	−24.4 (14.8)^d^	<.001	
FEV1/FVC	77.7 (6.6)	77.4 (6.5)	77 (6.5)	77.3 (6.5)	77.1 (6.5)	77.8 (6.5)	.73	

^a^Analysis of covariance was adjusted for sex, age, height, smoking habits, and physical activity (dichotomized: light and active [moderate and heavy]).

^b^FEV1: forced expiratory volume in 1 second (L).

^c^The presence of the same symbol in the same row indicates that there were no significant differences between pulmonary function and the number of components.

^d^The presence of the same symbol in the same row indicates that there were no significant differences between pulmonary function and the number of components.

^e^FEV1%: percentage of predicted forced expiratory volume in 1 second.

^f^FEV1 reduction (%) = ([FEV1 − expected FEV1]/FEV1) × 100.

^g^FVC: forced vital capacity (L).

^h^FVC%: percentage of predicted forced vital capacity.

^i^FVC reduction (%) = ([FVC − expected FVC]/FVC) × 100.

### Number of MetS Criteria and Lung Dysfunction

This effect on pulmonary function variables was reflected in the distribution of the prevalence of respiratory disorders. In other words, the alteration of the spirometric values detailed in the *MetS and Lung Function (Spirometric Parameters)*, *Individual MetS Criteria and Lung Function (Spirometric Parameters)* and *Number of MetS Criteria and Lung Function (Spirometric Parameters)* sections has clinical repercussions in the appearance of pathological respiratory patterns. In this respect, the proportion of participants who presented FEV1/FVC<0.7 (0: 8.9%, 1: 11.2%, 2: 12.7%, 3: 16.6%, 4: 21.1%, 5: 9.1%; *P*=.006) and FVC%<0.8 (0: 9.3%, 1: 14.4%, 2: 17.6%, 3: 28.7%, 4: 33.7%, 5: 45.5%; *P*<.001) was significantly higher among those with the most risk factors. This trend was also observed in the rates of lung dysfunction (0: 17.4%, 1: 23.6%, 2: 25.7%, 3: 38.1%, 4: 46.3%, 5: 45.5%; *P*<.001), in RLD (0: 8.5%, 1: 12.5%, 2: 13%, 3: 21.5%, 4: 25.3%, 5: 45.5%; *P*<.001), and in MLD (0: 0.7%, 1: 2%, 2: 4.6%, 3: 7.2%, 4: 8.4%, 5: 0%; *P*<.001) but not in OLD (0: 8.1%, 1: 9.2%, 2: 8.1%, 3: 9.4%, 4: 12.6%, 5: 9.1%; *P*=.79). Table 4 shows the prevalence ratios as a function of the number of components. The results show a significant linear increase in the prevalence ratio of all the pulmonary disorders studied with an increasing number of components (*P*<.001), except for OLD.

**Table 4:** Prevalence ratios for lung disorders according to the number of metabolic syndrome criteria.

Variables			Number of components, prevalence ratios (95% CI)													*P* value^a^			*P* value^b^
			0 (n=540)		1 (n=554)		2 (n=393)		3 (n=223)		4 (n=95)		5 (n=11)						
FEV1^c^/FVC^d^<0.7			1 (reference)^e^		1.26 (0.88-1.80)		1.43 (0.99-2.08)		1.87 (1.25-2.78)		2.37 (1.48-3.8)		1.02 (0.16-6.78)		.006			<.001	
FVC%^f^<0.8			1 (reference)		1.56 (1.12-2.18)		1.9 (1.35-2.66)		3.1 (2.22-4.34)		3.64 (2.47-5.35)		4.91 (2.43-9.88)		<.001			<.001	
**Lung dysfunction**			1 (reference)		1.36 (1.07-1.72)		1.48 (1.15-1.89)		2.19 (1.71-2.81)		2.66 (2-3.53)		3.13 (1.77-5.54)		<.001			<.001	
	RLD^g^	1 (reference)		1.46 (1.03-2.08)		1.52 (1.05-2.22)		2.53 (1.74-3.67)		2.97 (1.91-4.62)		5.34 (2.64-10.8)		<.001			<.001		
	OLD^h^	1 (reference)		1.13 (0.77-1.66)		1 (0.65-1.55)		1.16 (0.70-1.90)		1.55 (0.85-2.82)		1.12 (0.17-7.39)		.79			.33		
	MLD^i^	1 (reference)		2.68 (0.86-8.37)		6.18 (2.11-18.13)		9.69 (3.27-28.65)		11.37 (3.49-37.01)		0		<.001			<.001		

^a^Chi-square test.

^b^Linear trend.

^c^FEV1: forced expiratory volume in 1 second (L).

^d^FVC: forced vital capacity (L).

^e^Reference category in statistical analysis.

^f^FVC%: percentage of predicted forced vital capacity.

^g^RLD: restrictive lung disease.

^h^OLD: obstructive lung disease.

^i^MLD: mixed lung disease.

## Discussion

### Principal Findings

This study aimed to identify the association between MetS and changes in spirometric parameters and precisely determine whether a higher number of MetS components is related to a worse state of lung function.

The results show that the presence of MetS is associated with worse lung function. In this study, participants with MetS showed lower mean FEV1, FEV1%, FVC, and FVC%. These findings were confirmed when adjusting for different explanatory variables, showing a clear effect of MetS on different spirometric parameters. In the case of FEV1/FVC, although its mean was lower in the MetS group, this association was lost when adjusting for other independent variables. This trend has been evidenced in populations of different ethnicities across cross-sectional and longitudinal designs, although discrepancies in FEV1/FVC have been observed [31-36]. Kim et al [32] showed that after 6 years of follow-up, participants who had MetS at the beginning of the study or developed MetS during the study had a greater decline in FVC and FEV1 than those who were healthy. However, the difference was not significant in the case of FEV1. Ford et al [33] found that participants with MetS had lower FEV1, FEV1%, FVC, and FVC% and higher FEV1/FVC.

These changes in spirometric parameters have clinical relevance, as they translate into the development of lung dysfunction. However, there are inconsistencies in the type of alteration most present in participants with MetS. Some authors state that OLD is more prevalent [12,34,37], whereas others show that RLD is predominant [12,33,36,38-40]. The results of our study show a higher proportion of participants with FEV1/FVC<0.7 and FVC%<0.8 among those diagnosed with MetS. This was reflected in a higher prevalence of lung dysfunction, RLD, and MLD, with no difference observed in OLD. In contrast to our results, Buchman et al [34] found that among men and women aged >60 years with MetS, there was no higher prevalence of FEV1/FVC<0.7 compared with those without MetS. However, we did not analyze by age group, so we do not know what happens specifically in those aged >60 years. Scarlata et al [39] observed that participants with RLD had 3-fold higher odds of MetS (95% CI 1.16-7.89) than those with normal spirometry, similar to findings by other authors [33]. Inconsistencies in the obstructive pattern may be (1) because some studies do not consider the mixed pattern, which may increase the percentage of patients with an obstructive pattern [37] or (2) because of the noninclusion of the restrictive pattern in the study [34]. However, it seems clear that there is a greater tendency for the presence of RLD in people with MetS [12,33,36,40].

Despite the clear association of MetS with lung dysfunction, the pathophysiological process remains unclear. However, several mechanisms that could be crucial in the process have been described, such as insulin resistance and low-grade inflammation, both of which are present in MetS [17,18,41], which is reflected in the lungs through different markers [42]. Therefore, it seems reasonable to state that damage in other body systems, identified according to MetS risk factors, is associated with lung damage [41]. In this regard, our results have shown that each MetS component (WC, triglycerides, HDL, glucose, and high blood pressure) can significantly modify each spirometric parameter once adjusted for explanatory variables. This fact has also been evidenced by other authors [37,38,43,44].

The accumulation of systemic damage, measured by the number of MetS criteria, has been associated with more significant organ damage [45-48]. In this regard, we have shown that having a higher number of MetS criteria was related to a significantly worse lung function (spirometric parameters, except for FEV1/FVC), although no statistically significant differences were found between some analysis groups defined by the number of MetS criteria. In addition, linear percentage reductions of FVC and FEV1 were observed among the different groups. This inverse relationship has been observed in other populations, highlighting the association of central obesity with lung function decline [31,33,35-38,49,50].

These findings are essential for public health in any country. Therefore, it could be recommended that when health care professionals observe a progressive loss of lung function in a patient, they should be alerted and explore for possible cardiometabolic impairment. Concerning the latter, it is noteworthy that a significant linear increase in the prevalence ratios of pulmonary disorders was observed when the number of MetS components increased. Specifically, the decreases in FVC% below 80% and in the restrictive pattern stand out. In this regard, Lee et al [38] found a linear growth trend in the prevalence of RLD in men but not in women. Chen et al [50] reported a significant linear trend between the accumulation of MetS components and the decline in FVC% and FEV1% in both men and women. Similar results were found by Yoon et al [36]. They also reported that a significant increase in the odds ratio for RLD was observed with an increasing number of risk factors, with the magnitude varying according to the adjustment made. This trend was not observed for OLD [36].

Regardless of the type of lung disorder associated with MetS, several researchers have highlighted the need to intervene in these patients to ensure better respiratory health [12,32,33,50]. From our perspective and based on the results obtained, we believe that governments should promote public health programs that include the detection of cardiometabolic disorders and their impacts on lung health. Although some organizations consider the magnitude of the most critical public health problems as risk factors for lung disease [15,51,52], only a few consider metabolic disorders such as obesity [53]. The accumulation of visceral fat, the primary pathophysiological mechanism in the development of insulin resistance and thus of major metabolic disturbances, requires public health attention [54]. Therefore, metabolic disorders, such as MetS, should be included in the programs more concretely, as the evidence is increasingly strong regarding the relationship between metabolic disorders and lung disorders [55,56], even pointing to lung cancer [57].

With this inclusion, health professionals could be encouraged to perform routine spirometry in health examinations to detect early lung disorders, both those of both respiratory origin and those of cardiometabolic origin, not only in the working population (higher risk) but also in the general population [25]. Health professionals are on the frontline, and public health must provide strategies to diagnose and prevent lung disorders and protect and promote health [58], which has been advocated for years [59,60]. This idea gains relevance in a context where spirometry may be underused or, when used, misused [61,62].

### Limitations and Strengths

Owing to the epidemiological design, it is impossible to establish a cause-effect relationship between the increase in the number of cardiometabolic risk factors and the progressive alteration of spirometric variables. It would be interesting to conduct a retrospective cohort study to determine whether exposure to different cardiometabolic alterations modifies lung function and increases pulmonary disease incidence. Another possible limitation is the small number of participants in the group with 5 MetS criteria, which makes it difficult to analyze their lung function status. However, given the sample’s representativeness, we consider that its small size is linked to what can be found in real settings, where the presence of these individuals is scarce. Moreover, given the particular characteristics of the sample (workers, ie, the working population), extrapolation to the general population (more sedentary) is challenging. However, workers represent a significant proportion of the people, meaning that the participants analyzed cover an essential demographic stratum. Although workers’ smoking habits have been controlled, including the number of annual packs of cigarettes in data collection could improve the accuracy of the analysis. Despite the limitations, the robustness of the statistical tests, the control of confounding variables, and the large sample size make the results consistent. Furthermore, the methodology used has facilitated comparison with similar studies, making it possible to reinforce knowledge on the topic.

### Conclusions

The presence of a greater number of MetS criteria (cardiometabolic risk factors) has been associated with increased lung dysfunction and a higher prevalence of pulmonary impairments, especially those of the restrictive and mixed types. In addition, the findings show that the comorbid occurrence of risk factors leads to a deterioration of FVC and FEV1. The findings highlight the need for governments to consider the importance of cardiometabolic health in lung function when formulating public health policies that are to be implemented in hospitals, health centers, companies, etc. In this context, spirometry could be crucial for health professionals to monitor patients at a risk of developing chronic pathologies. In addition, routine spirometry helps detect changes in lung function related to cardiometabolic disorders. In short, spirometry is an accessible method that should be used to prevent and provide early care for diseases, including those not of pulmonary origin.

## Abbreviations

DM: diabetes mellitus
FEV1%: percentage of predicted forced expiratory volume in 1 second
FEV1: forced expiratory volume in 1 second
FVC%: percentage of predicted forced vital capacity
FVC: forced vital capacity
HDL: high-density lipoprotein
MD: mean difference
MetS: metabolic syndrome
MLD: mixed lung disease
OLD: obstructive lung disease
RLD: restrictive lung disease
WC: waist circumference

## Data Availability

The data sets generated and analyzed during this study are not publicly available because they belong to a public institution but are available from the corresponding author upon reasonable request.
